# Gram-Negative Bacterial Infections in Cardiac Implantable Electronic Devices: Insights from a Retrospective Analysis of Multidrug-Resistant and Non-Multidrug-Resistant Isolates

**DOI:** 10.3390/pathogens14030215

**Published:** 2025-02-22

**Authors:** Georgios Schinas, Rafail Koros, Ioannis Ntalakouras, Skevos Sideris, Angelos Perperis, Georgios Leventopoulos, Periklis Davlouros, Karolina Akinosoglou

**Affiliations:** 1 School of Medicine, University of Patras, 26504 Rio, Greece; georg.schinas@gmail.com (G.S.); levent2669@gmail.com (G.L.); pdav@upatras.gr (P.D.); 2 Division of Cardiology, University General Hospital of Patras, 26504 Rio, Greece; korosraf@hotmail.com (R.K.); angelosperperis@msn.com (A.P.); 3 Department of Cardiology, Ippokrateio General Hospital of Athens, 11527 Athens, Greece; ioantalak@gmail.com (I.N.); skevos1@otenet.gr (S.S.); 4 Department of Internal Medicine and Division of Infectious Diseases, University General Hospital of Patras, 26504 Rio, Greece

**Keywords:** cardiac implantable electronic devices, gram-negative bacterial infections, multidrug-resistant pathogens, device-related infections, cardiac rhythm management devices, antimicrobial resistance, device extraction

## Abstract

Cardiac implantable electronic device (CIED) infections caused by Gram-negative bacteria are uncommon but potentially life-threatening. This study examined patients with Gram-negative CIED infections, investigating the clinical characteristics of patients harboring multidrug-resistant (MDR), versus non-MDR, isolates. A retrospective observational analysis was conducted at two tertiary Greek University Hospitals from 2015 to 2020. Patients were identified through microbiological cultures from device-related sites (pocket, lead, generator), with infections classified as MDR or non-MDR based on antimicrobial susceptibility profiles. Comprehensive data were collected, including demographic characteristics, clinical parameters, procedural details—on both the last device procedure and subsequent extraction procedure—infection-related findings, and microbiological profiles. In total, 18 patients were identified, with an equal distribution of 9 MDR and 9 non-MDR cases. The study population had a median age of 78 years, with 33.3% female patients, and a median Charlson Comorbidity Index of four. *Pseudomonas aeruginosa* was the most prevalent isolated species. Comparative analysis revealed that MDR patients had higher median SOFA (Sequential Organ Failure Assessment) scores (2 vs. 0, *p* = 0.07), longer time to device extraction (50% vs. 88.9% extracted within one month, *p* = 0.079), and higher blood culture positivity (80% vs. 37.5%, *p* = 0.135). Despite similar demographic characteristics, MDR infections demonstrated more complex clinical profiles, with a trend towards increased disease severity.

## 1. Introduction

Cardiac implantable electronic devices (CIEDs), including pacemakers, implantable cardioverter-defibrillators (ICDs), and cardiac resynchronization therapy (CRT) devices, are increasingly utilized to manage a wide range of cardiac conditions [1]. Despite their significant benefits in reducing morbidity, and in some instances even mortality [2], these devices are susceptible to infections, which remain a challenging and potentially life-threatening complication with a consistently increasing incidence [3]. Reported one-year mortality rates vary widely, ranging from 5% to 20% [4,5]. Long-term survival is significantly impacted in patients with CIED infections, with an elevated risk of mortality persisting for up to three years post-infection, independent of demographics or comorbidities [6].

While Gram-positive bacteria, particularly *Staphylococcus aureus* and coagulase-negative staphylococci, are well-recognized pathogens in CIED infections, Gram-negative bacterial (GnB) infections are far less common, comprising approximately 10% of cases [7,8]. Common GnBs implicated in CIED infections include *Escherichia coli*, *Pseudomonas*, *Klebsiella*, and *Serratia species*, while less common organisms, such as *Burkholderia*, are also occasionally encountered [9]. In general, compared with CIED infections caused by Gram-positive bacteria, those due to GnBs are more likely to present as localized pocket infections and may be more common in patients with prior CIED infections or other comorbidities [10]. In some cases, and although rare [11,12], GnB-bacteremia originating from sources such as intra-abdominal or urinary tract infections may also seed the device hematogenously [13]. Once bacteria have adhered to the device surface, they initiate biofilm formation, which plays a central role in the persistence and treatment resistance of device-related infections [14].

Effective management of biofilm-associated infections caused by GnB often requires combined antibiotic therapy, aimed at achieving potent antibiofilm activity [15]. However, in cases where antibiotics alone are used for treatment, in-hospital mortality rates have been reportedly high [16]. Consequently, complete device removal, or extraction, is considered the gold standard for managing CIED infections effectively. The development of multidrug resistance (MDR) among GnB further complicates the choice of antimicrobial treatment, as resistance acquisition can variably alter biofilm production across different species, although no consistent differences in biofilm production between MDR and non-MDR isolates have been reported [17]. The emergence of MDR-GnBs in CIED infections thus presents a significant therapeutic challenge, limiting treatment options and potentially impacting clinical outcomes.

Although previous studies have explored the prevalence and clinical outcomes of CIED infections caused by GnB [10,18], clinical data specifically addressing MDR isolates are limited. This study presents a detailed analysis of GnB-CIED infections, comprising one of the most comprehensive case series to date on the clinical impact of MDR status. By sharing our experience in managing MDR GnB-CIED infections and comparing them with non-MDR cases, we aim to improve the understanding of the clinical and treatment challenges of these rare infections.

## 2. Materials and Methods

This study is a retrospective observational analysis aimed at evaluating the characteristics of Gram-negative infections associated with CIEDs, focusing on their clinical and microbiological profiles. Eligible patients were those with confirmed CIED infections due to Gram-negative pathogens, as identified through positive microbiological cultures. The study further aims to compare characteristics and outcomes between infections caused by MDR and non-MDR Gram-negative isolates. The research was conducted at the Cardiology Departments of two tertiary Greek University Hospitals, with data collection spanning patients admitted between 2015 and 2020. This study was conducted in compliance with the Declaration of Helsinki and approved by the Institutional Review Board (IRB) 267/29 April 2021. Given the retrospective nature, patient consent was waived.

### 2.1. Study Population

Eligible patients included adults (≥18 years) with confirmed CIED infections due to GnB, identified through positive microbiological cultures from CIED-related sites (pocket, lead, or generator). Inclusion required a comprehensive antimicrobial susceptibility profile to classify infections as MDR or non-MDR. Patients with polymicrobial infections involving both GnB and either Gram-positive or fungal organisms were included. Patients with non-Gram-negative CIED infections, evidence of contamination, or incomplete microbiological susceptibility data were excluded.

### 2.2. Data Collection

Data for each eligible patient was extracted from the institution’s electronic medical records, microbiology laboratory systems, and patient files. Demographic and baseline clinical characteristics included variables such as age, sex, Charlson Comorbidity Index (CCI), Sequential Organ Failure Assessment (SOFA) score, and immunosuppression status. Information on comorbidities and other relevant clinical conditions was also gathered.

Device and procedural data included information on both the last device-related procedure preceding the infection and the subsequent extraction procedure performed to control the infection. Variables concerning the last procedure captured details about the primary indication for the device implant, the type of device (defibrillator or pacemaker), the number of leads (i.e., single chamber pacemaker VR PPM or dual chamber pacemaker DR PPM), type of procedure (index or generator replacement), and placement site (left or right infraclavicular). Additional data collected included the presence of epicardial leads, anticoagulation status, history of prior pocket infections, use of an antibacterial envelope, and the Shariff score, to assess infection risk. The interval between the last procedure and the onset of infection was also documented. Infection-related findings that led to the extraction procedure were recorded, including imaging results such as the presence of vegetations on transthoracic or transesophageal echocardiography (TTE or TOE) and infection-related complications, including infective endocarditis, bacteremia, and septic embolism. Data on whether the infection diagnosis occurred within 90 days of the last procedure was also included.

Details regarding the subsequent extraction procedure were recorded, specifying the procedural method (transvenous or surgical) and the outcome, defined as either successful complete extraction or partial removal of infected components. Data on additional measures taken during the extraction, such as new device implantation with epicardial leads, were also collected. Following extraction, the total duration of hospital stays and post-operative complications were documented to assess in-hospital outcomes, including mortality and complications occurring during the hospitalization period.

### 2.3. Microbiological Data Collection

Microbiological data were drawn from cultures taken at sites relevant to the CIED infection (such as blood, leads, pocket, and generator), documenting the specific Gram-negative species identified and their respective susceptibility profiles. Documentation of polymicrobial presence and any concurrent bacteremia was also recorded. Laboratory-confirmed bloodstream infections were defined as previously described [19]. Contamination was defined as the presence of specific commensal or environmental organisms cultivated from a single blood culture set out of a series, which did not represent true bacteremia [20]. A list of common commensals was referenced from the CDC National Healthcare Safety Network Master Organism List, available at: https://professionals.wrha.mb.ca/ (accessed on 23 April 2024). 

In both study sites, Gram-negative pathogens were identified using VITEK^®^ 2 Gram-negative identification cards (bioMérieux, Craponne, France). The study assessed susceptibility to various antimicrobial agents, including ciprofloxacin, levofloxacin, gentamicin, amikacin, tobramycin, meropenem, imipenem, tigecycline, trimethoprim/sulfamethoxazole, and colistin. Minimum inhibitory concentrations (MICs) for ampicillin/sulbactam, ceftazidime, cefepime, piperacillin/tazobactam, and minocycline were analyzed for the respective study period. Antimicrobials were chosen based on guidelines from the European Committee on Antimicrobial Susceptibility Testing (EUCAST) and recommendations from the European Centre for Disease Prevention and Control (ECDC) and the Centers for Disease Control and Prevention (CDC). Results were interpreted following EUCAST standards in place at the time of testing, with isolates classified as susceptible, intermediate, or resistant. MICs for colistin were determined specifically using the broth microdilution method (SensiTest™ Colistin, Liofilchem, Italy), in accordance with EUCAST recommendations to ensure accuracy for this agent.

Multidrug resistance (MDR) was defined according to ECDC and CDC criteria as acquired non-susceptibility to at least one agent in three or more antimicrobial categories. Non-MDR isolates were those not meeting this definition. As no genotypic analysis was conducted, resistance mechanisms were inferred based on observed phenotypic resistance patterns.

### 2.4. Statistical Analysis

Descriptive analysis was performed to provide an overall characterization of Gram-negative CIED infections. Continuous variables were summarized as medians with interquartile ranges (IQR), while categorical variables were presented as frequencies and percentages. Continuous variables were compared using the Mann–Whitney U test. For categorical variables comparisons were performed using chi-square tests, or Fisher’s exact test. Data analysis was conducted using SPSS (v 29.0, IBM, Armonk, NY, USA), with statistical significance set at *p* < 0.002 after correction for multiple comparisons.

## 3. Results

### 3.1. Population Characteristics

This study included a total of 18 patients with Gram-negative CIED infections, as summarized in Table 1. The median age was 78 years (IQR: 61–87), and six patients (33.3%) were female. The cohort had a median CCI of 4 (IQR: 3–6), reflecting a range of comorbid conditions, though only one patient (5.6%) was immunosuppressed. The median hospitalization duration was 8 days (IQR: 4–10). There were no cases of in-hospital mortality observed in this study.

Bradyarrhythmia was the most common primary device indication, accounting for 12 cases (66.6%), followed by heart failure, and primary prevention of sudden cardiac death, each present in 2 cases (11.1%). The majority of patients, 11 (61.1%), had an ejection fraction (EF) > 50%, while 5 patients (27.7%) had an EF below 40%, indicating reduced cardiac function in this subset. Anticoagulation therapy was documented in seven patients (38.8%).

Procedural data revealed that 12 patients (66.7%) underwent device implantation as their last procedure prior to infection, while 3 patients (16.6%) had a recent box generator replacement. DRPPM was the predominant device type, used in 12 cases (66.7%), and all of these last procedures were performed transvenously. Regarding subsequent extraction outcomes, complete extraction was successful in 16 patients (88.9%), while partial lead extraction was required in 2 cases (11.1%).

Cultures from lead samples were positive in 13 cases (92.8%), and 7 patients (53.8%) had positive blood cultures. The percentages provided are proportional to the total number of cultures obtained. Additionally, seven infections (38.9%) were polymicrobial, with mixed Gram-positive and Gram-negative isolates documented in five cases (27.8%).

### 3.2. Microbiological Profile of Gram-Negative Isolates in CIED Infections

The distribution of primary Gram-negative isolates and their MDR status is shown in Table 2. Among the 18 isolates, 9 were classified as MDR. Of these, *Pseudomonas* spp. accounted for five of the nine isolates, followed by *Klebsiella* spp. (two of nine), *Proteus* spp. (one of nine), and *Serratia* spp. (one of nine). As for the *Pseudomonas* isolates, four exhibited extended-spectrum β-lactamase (ESBL) activity, while one additionally demonstrated resistance to carbapenems (CRPA). The *Klebsiella* isolates exhibited resistance to both carbapenems (CRE) and third-generation cephalosporins. Notably, their exclusive susceptibility to aztreonam suggested the potential presence of a metallo-β-lactamase enzyme (MBL). Both *Serratia* isolates displayed resistance to third-generation cephalosporins but remained susceptible to carbapenems. The *Proteus* isolate exhibited resistance to sulfamethoxazole-trimethoprim (SXT), quinolones, colistin, and tetracycline.

### 3.3. Comparison of MDR and Non-MDR Groups

Among the 18 patients with CIED infections included in this study, sensitivity testing identified an even distribution between those with MDR and non-MDR Gram-negative isolates (n = 9 per group). Comparative analyses of patient characteristics, procedural data, infection-related findings and outcomes between these two groups are presented in Table 3. Overall, demographic characteristics, hospitalization duration, CCI, device indication, ejection fraction, and procedural success rates were similar between groups, with no statistically significant differences noted.

SOFA scores were higher in the MDR group (median 2 [IQR: 1–2]) compared to the non-MDR group (median 0 [IQR: 0–1]), showing a trend toward significance (*p* = 0.07), suggesting greater disease severity at presentation in the MDR cohort. Additionally, the median time from diagnosis to extraction was shorter in the non-MDR group, with 88.9% of patients undergoing extraction within one month compared to 50% in the MDR group, approaching significance (*p* = 0.079). Blood culture positivity was more frequent in the MDR group (80%) than in the non-MDR group (37.5%), though this did not reach statistical significance (*p* = 0.135). Rates of polymicrobial infections and mixed polymicrobial infections were similar between groups, without statistically significant differences.

## 4. Discussion

This is the first study examining the clinical characteristics, microbiology, and procedural outcomes of MDR-GnB infections in CIED infections, offering important insights into the uncommon but clinically significant occurrence of GnB-CIED infections. There was no significant difference between the MDR-GnB and non-MDR-GnB groups in terms of epidemiology, presentation, and device related parameters. We have found that MDR GnB-CIED infections show a trend towards more severe disease at presentation, while respective device extraction takes longer than non-MDR GnB-CIED from time of diagnosis.

Gram-negative bacteria are uncommon but significant pathogens, which cause CIED infections. The current understanding of these infections is largely based on case reports and series [10,11,18,21,22]. The most extensive data available comes from Pascale et al., who performed a multicenter, international, retrospective, case–control study across 17 centers in Europe [18]. In total, 59 GnB-CIED infections over a 5-year period were included, in agreement with previous studies [7,10], confirming the low prevalence of these infections. Interestingly, neither study further compared to MDR and non-MDR GnB. Most infections were diagnosed 3 months after device implantation, potentially reflecting the fact that non-staphyloccocal pathogens appear to be less virulent, resulting in more protracted clinical manifestations before hospital admission [13]. In terms of clinical presentation, results vary. Previous studies have shown that GNB-CIED infections are more often associated with pocket infections compared to GPB-CIED infections [10], but this trend was not later confirmed [18]. We found no difference between groups in our report. Given the limited research on these infections, further studies are needed to better understand this aspect.

As far as microbial prevalence is concerned, in agreement with previous studies, *Pseudomonas* spp. predominated in our study, followed by various *Enterobacteriales* [10,13]. *Pseudomonas aeruginosa* was present in 38.8% of cases, with a high proportion of MDR strains (71.5%), compared to the pan-European study, which reported a lower overall prevalence (28.8%) and a 23.5% rate of extensively drug-resistant (XDR) strains [18]. *Klebsiella* spp. was the second most commonly encountered GnB in our study (16.7%), with one-third of the isolates classified as MDR, whereas in the pan-European cohort, all *Klebsiella* isolates (8.4% overall) were MDR. *Proteus mirabilis* was found in 11.1% of our cases, with 50% of the isolates exhibiting MDR, whereas the pan-European study reported *Proteus* spp. in 8.4%, but with 100% of the isolates classified as MDR. Overall, in the pan-European study, only 11 out of 59 isolated strains were classified as either MDR or XDR, whereas in our study, the incidence was significantly higher.

A MDR incidence of 50% was found, in line with the high MDR prevalence in the Greek setting, as reflected in previous studies [23,24] and a recent European Centre for Disease Prevention and Control (ECDC) report [25]. Since the late 2000s, Greece has been dealing with an endemic issue of multidrug-resistant pathogens in its hospital sector, primarily driven by carbapenem-resistant Gram-negative bacilli. Overall, the country has some of the highest antimicrobial resistance rates in Europe [25]. No data on MDR-GnB CIED has so far been available, even though the incidence of resistance has been increasing alarmingly over the last decade. Previous reports have highlighted rising methicillin resistance in the context of Gram-positive CIED infections [8]. Our study was performed prior to the COVID-19 pandemic, hence; in line with local epidemiological data, resistance trends are expected to increase further [26,27]. This may indicate the widespread inappropriate use of broad-spectrum antibiotics and suggest that a significant proportion of patients acquire these pathogens in healthcare settings, which carries important implications for empirical therapy. Reallocation of antimicrobial stewardship resources and infection control policies to COVID-19 wards, have contributed majorly to this entity [28].

The Shariff score is recognized as a predictor of the risk of GPB-CIED infection occurring in the months after device implantation [29,30,31]. Although, the Shariff score has demonstrated its predictive value in patients with GPB, its effectiveness was not confirmed in the group of patients with GnB-CIED infection [18]. Similarly, in our cohort, no difference was found between MDR and non-MDR pathogens. Nonetheless, MDR patients tend to present in a more severe condition than non-MDR patients, as reflected by a trend towards higher SOFA scores in our study. This comes in agreement with previous studies showing increased mortality rates in these patients [32,33,34], including patients with CIED infections [10,18,21]. The increased mortality risk associated with resistant pathogens is primarily attributed to the initial use of inappropriate antibiotic therapy, rather than increased virulence of the resistant organisms themselves [32,35,36,37]. In our report, this could be reflected in the longer times from diagnosis to extraction, possibly associated with multiple rounds of unsuccessful antibiotic therapy. In this setting, delay or underestimation in diagnosis could also be responsible for high mortality rates. The FDG PET/CT was introduced in the 2015 ESC Criteria as a diagnostic tool for infections involving CIEDs [38,39,40]. Current evidence suggests that FDG PET/CT demonstrates a higher diagnostic yield for GnB-CIED infections, compared to those caused by GPB. Notably, in this study, among imaging modalities used for diagnosing CIED infections, echocardiography was the most commonly employed. This indicates that the prevalence of GNB-associated CIED infections in this cohort and in broader clinical practice may have been underestimated due to the limited diagnostic efficiency of traditional methods. These findings underscore the importance of rapid identification and appropriate treatment of resistant infections to mitigate mortality risks.

No periprocedural factors appear to be significantly related to MDR-GnB in the current study. Although a trend towards higher prevalence of MDR-GnB, when implantation was performed via the right subclavian vein, was noted, this was not significant. Similarly, unlike previous studies reporting a relation between GnB-CIED infection and the type of device used [18,41,42], the latter did not seem to be associated with pathogen resistance patterns. Moreover, in our study, antimicrobial envelopes were not utilized. While various preventive measures against biofilm formation on implanted medical devices have been explored, the specific application of these strategies in the context of CIEDs remains limited. Currently, the primary focus has been on the use of antimicrobial envelopes, such as the AIGISRx^®^ antimicrobial envelope (TyRX Inc., Monmouth Junction, NJ, USA), which have shown promise in reducing the risk of infections associated with CIEDs [43,44]. A meta-analysis of 14,859 procedures further confirmed these findings, demonstrating that antibiotic envelopes reduced the risk of all infections by 59% (RR: 0.41, 95% CI: 0.31–0.54, *p* < 0.05) and major infections by 52% (RR: 0.48, 95% CI: 0.32–0.70, *p* < 0.05) [45]. Additionally, the relative cost-effectiveness of antimicrobial envelopes has been well-documented [46]. These envelopes are designed to release antibiotics like rifampin and minocycline locally at the implantation site, effectively combating bacterial colonization and biofilm formation—key contributors to device-related infections. In addition to antimicrobial envelopes, future research could explore other strategies that have proven successful in different medical contexts, such as the placement of antibiotic impregnated beads, which could help salvage device extraction in cases where it is not feasible [47].

This study has several limitations inherent to its retrospective observational design. First, reliance on medical records and microbiological data may introduce information bias, as not all clinical and procedural details may have been consistently documented or recorded. In this context, diagnostic uncertainty in CIED infection can be challenging, especially among patients who present with positive blood cultures without generator pocket findings, despite established EHRA criteria [48]. The relatively small sample size, particularly in each subgroup, limits the statistical power to detect subtle differences between the MDR and non-MDR groups, and may restrict the generalizability of findings. Additionally, as the study is limited to one country, the results may reflect institutional-specific practices and resistance patterns, which may not be representative of other settings. Moreover, the inclusion of polymicrobial infections complicates the interpretation of outcomes specific to Gram-negative pathogens, as co-infecting organisms may influence disease progression and clinical response. Controlling for this and other confounders through advanced analyses was not feasible due to the small sample size. Furthermore, our study lacks detailed information on prophylaxis, hospitalization, and prior or current antibiotic therapy for these patients. Another limitation is the lack of surveillance colonization cultures, which precludes an assessment of MDR colonization of the skin among participants. Colonization is a known risk factor for subsequent bacteremia [49], and future studies should consider evaluating colonization patterns. Lastly, the absence of long-term follow-up data restricts our ability to assess recurrence rates and long-term clinical outcomes; we recommend that future studies incorporate extended follow-up to address these critical aspects.

Despite these limitations, our study provides valuable insights into the clinical characteristics, microbiology, and procedural outcomes of MDR-GnB infections in CIEDs, shedding light on this rare yet clinically significant entity. While Gram-positive infections are more commonly encountered in CIED-related infections, our findings highlight the emerging challenge of Gram-negative pathogens, particularly MDR strains, which may present with more severe disease and longer treatment times compared to non-MDR strains. Continued research is essential to further explore the epidemiology and management of these infections in comparison to the more prevalent Gram-positive cases.

## Figures and Tables

**Table 1:** Population Characteristics.

Population Characteristics	Median (IQR)/Count (%)
*Demographics and Baseline Characteristics*	
Female Sex (%)	6 (33.3)
Age (years)	78 (61–87)
Charlson Comorbidity Index (CCI)	4 (3–6)
SOFA Score	1 (0–2)
Immunosuppression Status (Yes)	1 (5.6)
In-Hospital Mortality (Yes)	0
*Last Procedure Characteristics*	
Device Primary Indication	
Bradyarrhythmia	12 (66.6)
Heart Failure	2 (11.1)
Primary Prevention	2 (11.1)
Secondary Prevention	1 (5.6)
Unknown	1 (5.6)
Ejection Fraction (EF) Category	
50%	11 (61.1)
40–50%	1 (5.6)
<40%	5 (27.7)
Unknown	1 (5.6)
Anticoagulation Status (Yes)	7 (38.8)
Type of Last Procedure	
Index Procedure	12 (66.7)
Generator Replacement	3 (16.6)
Unknown	3 (16.6)
Device Type	
DRPPM	12 (66.7)
DRICD	2 (11.1)
VRICD	2 (11.1)
CRTD	1 (5.6)
VDDPPM	1 (5.6)
Presence of Epicardial Lead (Yes)	0 (0)
Implantation Site	
Left Infraclavicular	12 (66.7)
Right Infraclavicular	6 (33.3)
Antibacterial Envelope Use (Yes)	0
Shariff Score	
0	3 (16.7%)
1	6 (33.3%)
2	3 (16.7%)
3	4 (22.2%)
4	1 (5.6%)
Unknown	1 (5.6%)
Previous Pocket Infection (% Yes)	6 (33.3)
*Infection-Related Findings*	
Infective Endocarditis (Yes)	6 (33.3)
Septic Embolism (Pulmonary/Other/No)	0
TTE Vegetations (Number, Site)	
1 Vegetation	1 (5.6) Vegetation on lead
2 Vegetations	3 (16.7) 2 cases: 1 vegetation on lead, 1 on valve 1 case: 2 vegetations on valves
No Vegetations	13 (72.2)
Unknown	1 (5.6)
TOE Vegetations (Number, Site)	
1 Vegetation	0 (0)
2 Vegetations	1 (5.6) 2 vegetations on valves
No Vegatations	2 (11.1)
Unknown	15 (83.3)
*Extraction Procedure*	
Time Since Last Implant/Box Change (days)	26 (11–73)
Time From Diagnosis to Extraction (months)	
<1 Month	12 (70.6)
3 Months	5 (29.4)
Procedure Type	
Transvenous	18 (100)
Procedure Outcome	
Success	16 (88.9)
Partial Lead Extraction	2 (11.1)
New Implantation (Yes-epicardial lead)	2 (11.1)
Total Leads (Including Abandoned)	
1	3 (16.7)
2	13 (72.2)
3	2 (11.1)
Abandoned Leads (number)	
0	16 (88.9)
1	2 (11.1)
Postoperative Hematoma (Yes)	1 (5.6)
Temporary Pacemaker (TPM) (Yes)	0
*Microbiology and Culture Results*	
Culture Sample Positivity (n [Culture Positivity%/Number of Cultures Obtained])	
Blood (n = 13)	5 (27.8)
Leads (n = 14)	13 (92.8)
Pocket (n = 7)	7 (100)
Generator (n = 1)	1 (100)
Polymicrobial Infection (Yes)	7 (38.9)
Multi-Drug-Resistant spp. (Yes)	9 (50%)

IQR: interquartile range; CCI: Charlson Comorbidity Index; SOFA: Sequential Organ Failure Assessment; EF: ejection fraction; DR PPM: dual chamber pacemaker; DR ICD: dual chamber implantable cardiac defibrillator, VR ICD: single chamber (ventricle) implantable cardiac defibrillator; CRTD: Cardiac Resynchronization Therapy Defibrillator; PMVDD: Permanent Medtronic Ventricular Demand Device; TTE: Transthoracic Echocardiography; TOE: transesophageal echocardiography; TPM: Temporary Pacemaker; spp.: species.

**Table 2:** Primary Isolated Species.

Primary Isolated Species	Total Count (%)	MDR Status Distribution (%)
*Enterobacter cloacae*	1 (5.6)	Non-MDR: 100%
*Escherichia coli*	2 (11.1)	Non-MDR: 100%
*Klebsiella* spp.	3 (16.7)	MDR: 33.3%, Non-MDR: 66.7%
*Pseudomonas aeruginosa*	7 (38.8)	MDR: 71.5%, Non-MDR: 28.5%
*Proteus mirabilis*	2 (11.1)	MDR: 50%, Non-MDR: 50%
*Serratia marcescens*	2 (11.1)	MDR: 100%
*Sphingomonas paucimobilis*	1 (5.6)	Non-MDR: 100%
Total	18 (100)	MDR: 50%, Non-MDR: 50%

**Table 3:** MDR Subgroup Comparison.

MDR Subgroup Comparison	MDR Group (n = 9)	Non-MDR Group (n = 9)	*p*-Value
*Demographics and Baseline Characteristics*	Median (IQR)/Count (%)		
Age (years)	82 (75–92)	71 (58–85)	0.258
Female Sex (%)	2 (22.2%)	4 (44.4%)	0.11
Charlson Comorbidity Index (CCI)	5 (4–7)	4 (3–5)	0.37
SOFA Score	2 (1–2)	0 (0–1)	0.07
*Device and Last Procedure Characteristics*			
Ejection Fraction (EF) Category			0.539
<40%	2 (22.2%)	3 (33.3%)	
>50%	5 (55.6%)	6 (66.7%)	
40–50%	1 (11.1%)	0 (0%)	
Anticoagulation Status (Yes)	3 (33.3%)	4 (44.4%)	0.629
Type of Last Procedure			0.194
Index Procedure	7 (77.8%)	5 (55.6%)	
Generator Replacement	1 (11.1%)	2 (22.2%)	
Time Since Last Implant/Box Change (days)	36 (12–80)	13 (4–184)	0.613
Device Type			0.176
DRPPM	7 (77.8%)	5 (55.6%)	
DRICD	0 (0%)	2 (22.2%)	
VRICD	0 (0%)	2 (22.2%)	
CRTD	1 (11.1%)	0 (0%)	
Site of Implantation			0.317
Left infraclavicular	5 (55.6%)	7 (77.8%)	
Right infraclavicular	4 (44.4%)	2 (22.2%)	
Shariff Score	1 (0–3)	2 (1–3)	0.236
Previous Pocket Infection (Yes)	3 (33.3%)	3 (33.3%)	1
*Infection-Related Findings*			
Diagnosis < 90 Days from Procedure			0.599
Yes	3 (33.3%)	2 (22.2%)	
No	6 (66.7%)	7 (77.8%)	
Infective Endocarditis (Yes)	4 (44.4%)	2 (22.2%)	0.317
TTE Vegetations			0.99
1 Vegetation	0	1 (11.1%)	
2 Vegetations	2 (22.2%)	1(11.1%)	
No Vegetations	6 (66.7%)	7 (77.8%)	
Unknown	1 (11.1%)	0	
TOE Vegetations			1
1 Vegetation	0	0	
2 Vegetations	1 (11.1%)	0	
No Vegetations	1 (11.1%)	1 (11.1%)	
Unknown	6 (66.7%)	8 (88.9%)	
*Extraction Procedure*			
Time Since Last Implant/Box Change (days)	36 (12–80)	13 (4–184)	0.61
Extraction Outcome (Success)	8 (88.9%)	8 (88.9%)	0.381
Partial Extraction (Yes)	1 (11.1%)	1 (11.1%)	1
Time from Diagnosis to Extraction			0.079
<1 Month	4 (50%)	8 (88.9%)	
3 Months	4 (50%)	1 (11.1%)	
*Microbiology and Culture Results*	Culture Site Positivity (n [Culture Positivity%/Number of Cultures Obtained])		
Blood Culture (Positive/Negative)			0.135
No	1 (20%)	5 (62.5%)	
Yes	4 (80%)	3 (37.5%)	
Leads (Presence/Absence)			0.439
No	0 (0%)	1 (11.1%)	
Yes	5 (100%)	8 (88.9%)	
Pocket Infection (Yes/No)			
Yes	6 (100%)	1 (100%)	No *p*-value (constant)
Generator Infection (Yes/No)			
Yes	1 (100%)	0 (0%)	No *p*-value (single case)
Polymicrobial Isolates (Yes)	3 (33.3%)	4 (44.4%)	0.629

MDR: multi-drug resistant; CCI: Charlson Comorbidity Index; SOFA: Sequential Organ Failure Assessment; EF: ejection fraction; DR PPM: dual chamber pacemaker; DR ICD: dual chamber implantable cardiac defibrillator; VR ICD: single chamber (ventricle) implantable cardiac defibrillator; CRTD: Cardiac Resynchronization Therapy Defibrillator; TTE: Transthoracic Echocardiography; TOE: transesophageal echocardiography.

## Data Availability

Data can be made available upon reasonable request.
